# Periodontal Disease Is Associated With Increased Risk of Hypertension: A Cross-Sectional Study

**DOI:** 10.3389/fphys.2019.00440

**Published:** 2019-04-25

**Authors:** Ming-Juan Zhao, Yi-Xin Qiao, Lan Wu, Qiao Huang, Bing-Hui Li, Xian-Tao Zeng

**Affiliations:** ^1^Center for Evidence-Based and Translational Medicine, Zhongnan Hospital of Wuhan University, Wuhan, China; ^2^Department of Evidence-Based Medicine and Clinical Epidemiology, The Second Clinical College of Wuhan University, Wuhan, China; ^3^Center for Evidence-Based Medicine, Institute of Evidence-Based Medicine and Knowledge Translation, Henan University, Kaifeng, China; ^4^Department of Cardiology, The First Affiliated Hospital of Henan University, Kaifeng, China; ^5^Department of Innovation Laboratory, The Affiliated High School of Henan University, Kaifeng, China; ^6^Department of Stomatology, Zhongnan Hospital of Wuhan University, Wuhan, China

**Keywords:** blood pressure, hypertension, periodontal disease, periodontitis, risk factor, inflammatory disease

## Abstract

**Objective:** Published evidence showed that periodontal disease is associated with hypertension. However, relevant findings remain controversial, with few evidences focusing on Chinese population. Therefore, the aim of this study was to investigate the association between periodontal disease and hypertension in Chinese population.

**Methods:** A total of 4,930 participants from an available health examination that was carried out in 2017 were selected for this retrospective study. The correlations between periodontal disease and hypertension were investigated using univariate and multiple logistic regression analyses and propensity score adjusted analysis. Interaction and subgroup analyses were also used to detect variable factors.

**Results:** Finally, a total of 3,952 participants aged 30–68 years were eligible for this study. The results showed that hypertension risk was statistically significant associated with periodontal disease either in unadjusted (OR = 1.28, 95%CI = 1.14–1.47) or in adjusted (OR = 1.34, 95%CI = 1.14–1.58) model. Result from propensity score adjusted analysis also demonstrated a similar association (OR = 1.23, 95%CI = 1.06–1.42).

**Conclusion:** Periodontal disease is significantly and positively correlated with increased risk of hypertension in Chinese population, and exact mechanisms of this association should be explored in future.

## Introduction

Periodontal disease is a complex polymicrobial inflammation, including gingivitis and periodontitis. According to the 2015 Global Burden of Disease (GBD) study, the prevalence of severe chronic periodontitis in 2015 has reached 616 million (Kassebaum et al., 2017). In China, the periodontal disease standardized DALYs rate has risen from 24.7 in 1990 to 25.7 in 2013 according to the data from 2013 GBD study (Zhang et al., 2017). Periodontal disease is considered as a potential risk factor for various systematic diseases, and previously published systematic reviews and meta-analyses have indicated that this disease could increase the risk of atherosclerotic complications, such as cardiovascular disease (Mustapha et al., 2007; Zeng et al., 2017), arterial stiffness (Schmitt et al., 2015), carotid intima-media thickness (Orlandi et al., 2014), carotid atherosclerosis (Zeng et al., 2016a), stroke (Lafon et al., 2014), coronary heart disease (Leng et al., 2015), erectile dysfunction (Cheraghi and Doosti-Irani, 2017), and hypertension (Martin-Cabezas et al., 2016). Hypertension is a well-known risk factor for atherosclerotic vascular diseases, with prevalence among adults of 34% in the United States (Roger et al., 2012) and 30–45% in European countries (Mancia et al., 2014). In China, hypertension is also a major public health burden, showing an adjusted prevalence of 29.6% [95% confidence interval (CI) = 28.9–30.4%] (Wang et al., 2014).

Many published epidemiological studies have investigated shared risk factors between periodontal disease and hypertension, such as age, gender, smoking, educational level, socioeconomic status, obesity and diabetes (Tsioufis et al., 2011). And multiple populations have been targeted in such researches, including those from Poland, Korea, Japan, France, Malaysia, Finland, Greece, United States, Puerto Rico, India, Tanzania, Brazil, Netherlands, Sweden, and Jordania (Tsioufis et al., 2011; Martin-Cabezas et al., 2016); the meta-analysis by Martin-Cabezas et al. (2016) suggested that periodontal disease significantly increased the risk of hypertension by 1.50 times [odds ratio (OR) = 1.50, 95%CI = 1.27–1.78]. Obviously, epidemiological data on Chinese population is still missing. Considering high prevalence and insufficient awareness of periodontal disease (Lin et al., 2014; Zhang et al., 2017) and hypertension (Martin-Cabezas et al., 2016) in China, we performed this cross-sectional study in order to provide evidence demonstrating the relationship between the two diseases in Chinese population.

## Materials and Methods

### Study Design and Data Extraction

With a retrospective design, this study adopted all available data from health examinations in Henan University which were carried out in 2017. We first identified 4,930 records with full information on name, gender, date of birth, relevant physical examination and laboratory examination. Records would be included for further analysis if information on gender, age (>30 years), periodontal status, weight, height, systolic blood pressure (SBP; mmHg), diastolic blood pressure (DBP; mm Hg), and fasting blood glucose (FBG, ng/mL) was all contained. All eligible data were finally divided into hypertensive and normotensive groups according to the presence or absence of hypertension. This study was reviewed and approved by the Committee for Ethical Affairs of the Huaihe Hospital of Henan University, Henan Province (Approval No. 2018068).

### Assessment of Variables

Systolic blood pressure and diastolic blood pressure were measured at participants’ right arm adopting a seated position after at least 5 min of resting using a standard mercury sphygmomanometer. The measurement repeated after 2 min after. The definition for hypertension referred to SBP ≥140 mm Hg, DBP ≥90 mm Hg or taking anti-hypertensive drugs.

Clinical oral examinations on participants seating in a dental chair were performed by dentists using a headlamp, a mouth mirror, and a periodontal probe. Periodontal status was assessed using the Community Periodontal Index of Treatment Needs (CPITN) according to the WHO guidelines (Cutress et al., 1987). For data analysis, periodontal status was divided into normal (CPI score of 0), periodontal disease (CPI score of 1–4), and periodontitis (CPI score of 3–4) groups.

The selection of covariates was conducted based on original recorded data and current knowledge of potential determinants of hypertension and periodontitis. Covariates that were used in analyses included age, gender, body mass index (BMI; kg/m^2^), FBG, serum lipid composition [total cholesterol (TC; mmol/L), triglycerides (TG; mmol/L), high-density lipoprotein cholesterol (HDL-C; mmol/L), low-density lipoprotein cholesterol (LDL-C; mmol/L)], erythrocyte sedimentation rate (ESR; mm/h), C-reactive protein (CRP; mg/L), urea nitrogen (UN; mmol/L), uric acid (UA; μmol/L), creatinine (μmol/L), and proteinuria.

### Statistical Analysis

Basic characteristics were summarized for both normotensive and hypertensive groups. Categorical variables were showed as count (percentage), while continuous ones as mean pulse/minus standard deviation or median (the first quartile, the third quartile) based on normal distribution test. Comparisons between normotensive and hypertensive groups were conducted using two independent sample *t*-test (or Wilcoxon rank sum test) for continuous variables and chi-squared test (or Fisher exact test) for categorical ones.

The association between periodontal disease and hypertension was examined with univariable and multivariable logistic regressions. Basic characteristics were adopted for univariable analysis while those with *p*-value less than 0.1 for multivariable analysis. Further and detailed evaluation was performed via additional logistic regression models for periodontal disease and periodontitis groups, individually. Four models were fitted for each part. Specifically, model 1 was constructed without adjustment; model 2 was constituted with adjustment for gender, age, BMI, FBG, TC, TG, LDL-C, and HDL-C; model 3 was added additional adjustment for ESR, CRP, UN, UA, creatinine, and proteinuria based on model 2. Model 4 employed covariate adjustment using propensity score for robustness evaluation. Statistical presentation included OR, 95% CI and corresponding *p*-value for simplify.

Interaction and subgroup analyses were operated to detect moderator effects. *P*-value for interaction was estimated via likelihood ratio test. The result was presented as forest plot. All statistical analyses were calculated with SAS 9.4 software. Tests on statistical significance were two-sided, and *p* < 0.05 was considered as significant level.

## Results

### General Characteristics

After subject selection, records on 3,952 participants aged 30–68 years were enrolled for this analysis. Of the participants, 2,761 were normotension and 1,191 were hypertension; 2,564 were free from periodontal disease, 1,388 had periodontal disease and 403 had periodontitis. The prevalence of periodontal disease was 35.12%. The median age was 43.00 (36.00–56.00) years in normotensive group and 57.00 (46.00–68.00) years in hypertensive group. The median BMI was 23.51 (21.47–25.51) kg/m^2^ in normotensive group and 25.31 (23.23, 27.43) kg/m^2^ in hypertensive group. Baseline characteristics of all these participants are presented in Table 1.

**Table 1:** Baseline characteristics of all participants (*N* = 3952).

Characteristics	Normotension (*n* = 2761)	Hypertension (*n* = 1191)	*P*-value
Gender (%)			<0.001
Male	1363 (49.37%)	834 (70.03%)	
Female	1398 (50.63%)	357 (29.97%)	
Age (years)^∗^	43.00 (36.00, 56.00)	57.00 (46.00, 68.00)	<0.001
Body mass index (kg/m^2^)^∗^	23.51 (21.47, 25.51)	25.31 (23.23, 27.43)	<0.001
Fasting blood glucose (ng/mL)^∗^	5.10 (4.80, 5.50)	5.40 (5.10, 6.00)	<0.001
Total cholesterol (mmol/L)^∗^	4.54 (3.98, 5.14)	4.72 (4.18, 5.41)	<0.001
Triglycerides (mmol/L)^∗^	1.07 (0.76, 1.58)	1.37 (0.93, 1.97)	<0.001
LDL-C (mmol/L)^∗^	2.60 (2.17, 3.13)	2.81 (2.35, 3.29)	<0.001
HDL-C (mmol/L)^∗^	1.26 (1.11, 1.43)	1.24 (1.10, 1.43)	0.482
ESR (mm/h)^∗^	5.00 (3.00, 10.00)	6.00 (3.00, 11.00)	0.017
C-reactive protein (mg/L)^∗^	0.60 (0.40, 1.30)	1.00 (0.60, 2.00)	<0.001
Urea nitrogen (mmol/L)^∗^	4.60 (3.90, 5.40)	4.90 (4.20, 5.80)	<0.001
Uric acid (μmol/L)^∗^	262.80 (212.15, 320.95)	301.50 (249.50, 354.20)	<0.001
Creatinine (μmol/L)^∗^	72.05 (62.85, 82.75)	74.80 (64.20, 85.50)	<0.001
Proteinuria (%)			<0.001
Negative	2576 (96.59%)	1044 (91.98%)	
Suspicious	63 (2.36%)	48 (4.23%)	
Positive	28 (1.05%)	43 (3.79%)	


*LDL-C, low-density lipoprotein cholesterol; HDL-C, high-density lipoprotein cholesterol; ESR, erythrocyte sedimentation rate; ^∗^, median (the first quartile, the third quartile).*

### Overall Results

Table 2 demonstrates the results from univariate and multivariate logistic regression analyses and propensity score adjusted model analysis. In univariate analysis, we uncovered that periodontal disease significantly increased hypertension risk by 1.28 times (OR = 1.28, 95%CI = 1.14–1.47), a similar result was obtained in periodontitis group (OR = 1.81, 95%CI = 1.45–2.26). Adjusted model 2 analysis found periodontal disease as a whole (OR = 1.33, 95%CI = 1.14–1.57) and periodontitis (OR = 1.28, 95%CI = 1.00–1.64) all significantly increased hypertension risk (all *p* < 0.05). Adjusted model 3 analysis also achieved similar results (periodontal disease: OR = 1.34, 95%CI = 1.14–1.58; periodontitis: OR = 1.29, 95%CI = 1.00–1.65; all *p* < 0.05). When we used propensity score adjusted model, the results were similar to those from multivariate logistic regression analyses (periodontal disease: OR = 1.23, 95%CI = 1.06–1.42; periodontitis: OR = 1.27,95%CI = 1.00–1.62; all *p* < 0.05).

**Table 2:** Multivariable analysis results of relationship between periodontal disease and hypertension.

Model	Periodontal disease		Periodontitis	
	OR (95% CI)	*p*-value	OR (95% CI)	*p*-value
Model 1	1.28 (1.11–1.47)	<0.001	1.81 (1.45–2.26)	<0.001
Model 2	1.33 (1.14–1.57)	<0.001	1.28 (1.00–1.64)	0.047
Model 3	1.34 (1.14–1.58)	<0.001	1.29 (1.00–1.65)	0.048
Model 4	1.23 (1.06–1.42)	0.006	1.27 (1.00–1.62)	0.048


*OR, odds ratio; CI, confidence interval. Model 1: unadjustment. Model 2: adjusted for age, gender, body mass index, fasting blood glucose, total cholesterol, triglycerides, low-density lipoprotein cholesterol, and high-density lipoprotein cholesterol based on model 1. Model 3: adjusted for erythrocyte sedimentation rate, C-reactive protein, urea nitrogen, uric acid, creatinine, and roteinuria based on model 2. Model 4: propensity score adjusted model.*

### Interaction and Subgroup Results

**FIGURE 1:**
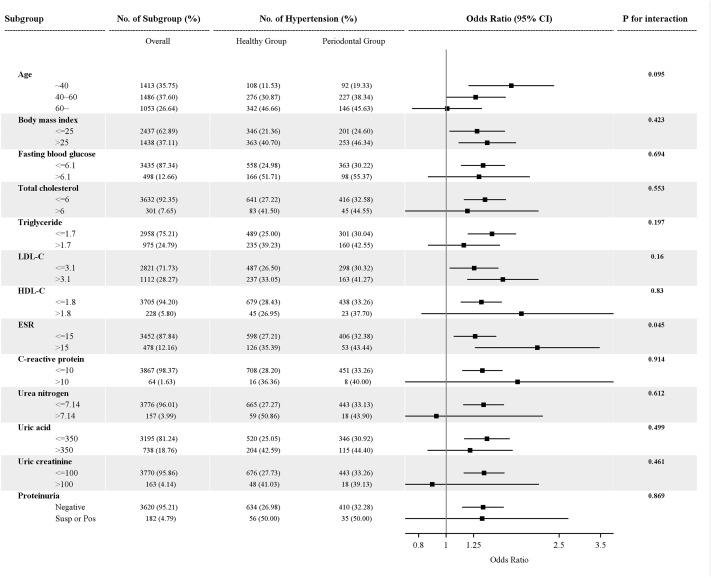
Results of interaction and subgroup analyses of relationship between periodontal disease and hypertension. LDL-C, low-density lipoprotein cholesterol; HDL-C, high-density lipoprotein cholesterol; ESR, erythrocyte sedimentation rate; CI, confidence interval; Susp or pos, suspicious or positive. *p* for interaction was test by likelihood ratio test.

Table 3 and Figure 1 presented the results from interaction and subgroup analyses. According, periodontal disease significantly increased hypertension risk in age <40 years group (OR = 1.69), BMI normal (OR = 1.29) and abnormal groups (OR = 1.40), FBG normal group (OR = 1.35), TC normal group (OR = 1.37), TG normal group (OR = 1.45), LDL-C normal (OR = 1.25) and abnormal groups (OR = 1.58), HDL-C normal group (OR = 1.33), ESR normal (OR = 1.26) and abnormal groups (OR = 2.09), CRP normal group (OR = 1.34), UN normal group (OR = 1.35), UA normal group (OR = 1.35), creatinine normal group (OR = 1.36) and proteinuria negative group (OR = 1.35), with *p* < 0.05 for all. And significant difference was observed in ESR (*p* = 0.045).

**Table 3:** Interaction and subgroup analyses results of relationship between periodontal disease and hypertension.

Subgroups	Samples (%)	Hypertension (%)		OR (95% CI)	*p*-value	*p* for interaction^∗^
		Healthy periodontium	Periodontal disease			
**Age (years)**						0.095
<40	1413 (35.75%)	108 (11.53%)	92 (19.33%)	1.694 (1.196–2.398)	0.0030	
40∼60	1486 (37.60%)	276 (30.87%)	227 (38.34%)	1.270 (0.995–1.621)	0.0553	
>60	1053 (26.64%)	342 (46.66%)	146 (45.63%)	1.007 (0.758–1.336)	0.9636	
**Body mass index (kg/m^2^)**						0.423
≤25	2437 (62.89%)	346 (21.36%)	201 (24.60%)	1.285 (1.025–1.611)	0.0297	
>25	1438 (37.11%)	363 (40.70%)	253 (46.34%)	1.395 (1.104–1.763)	0.0052	
**Fasting blood glucose (ng/mL)**						0.694
≤6.1	3435 (87.34%)	558 (24.98%)	363 (30.22%)	1.347 (1.126–1.612)	0.0011	
>6.1	498 (12.66%)	166 (51.71%)	98 (55.37%)	1.303 (0.861–1.971)	0.2099	
**Total cholesterol (mmol/L)**						0.553
≤6	3632 (92.35%)	641 (27.22%)	416 (32.58%)	1.369 (1.154–1.623)	0.0003	
>6	301 (7.65%)	83 (41.50%)	45 (44.55%)	1.188 (0.667–2.115)	0.5583	
**Triglycerides (mmol/L)**						0.197
≤1.7	2958 (75.21%)	489 (25.00%)	301 (30.04%)	1.448 (1.190–1.794)	0.0002	
>1.7	975 (24.79%)	235 (39.23%)	160 (42.55%)	1.154 (0.861–1.547)	0.3382	
**Low-density lipoprotein cholesterol (mmol/L)**						0.160
≤3.1	2821 (71.73%)	487 (26.50%)	298 (30.32%)	1.254 (1.028–1.529)	0.0258	
>3.1	1112 (28.27%)	237 (33.05%)	163 (41.27%)	1.582 (1.185–2.112)	0.0019	
**High-density lipoprotein cholesterol (mmol/L)**						0.83
≤1.8	3705 (94.20%)	679 (28.43%)	438 (33.26%)	1.329 (1.124–1.570)	0.0009	
>1.8	228 (5.80%)	45 (26.95%)	23 (37.70%)	1.84 (0.817–4.142)	0.141	
**Erythrocyte sedimentation rate (mm/h)**						0.045
≤15	3452 (87.84%)	598 (27.21%)	406 (32.38%)	1.264 (1.063–1.502)	0.008	
>15	478 (12.16%)	126 (35.39%)	53 (43.44%)	2.093 (1.261-3.472)	0.0043	
**C–reactive protein (mg/L)**						0.914
≤10	3867 (98.37%)	708 (28.20%)	451 (33.26%)	1.34 (1.137–1.580)	0.0005	
>10	64 (1.63%)	16 (36.36%)	8 (40.00%)	1.783 (0.298–10.671)	0.5263	
**Urea nitrogen (mmol/L)**						0.612
≤7.14	3776 (96.01%)	665 (27.27%)	443 (33.13%)	1.354 (1.146–1.599)	0.0004	
>7.14	157 (3.99%)	59 (50.86%)	18 (43.90%)	0.922 (0.388–2.191)	0.8545	
**Uric acid (μmol/L)**						0.499
≤350	3195 (81.24%)	520 (25.05%)	346 (30.92%)	1.391 (1.155–1.674)	0.0005	
>350	738 (18.76%)	204 (42.59%)	115 (44.40%)	1.214 (0.859–1.715)	0.2716	
**Creatinine (μmol/L)**						0.461
≤100	3770 (95.86%)	676 (27.73%)	443 (33.26%)	1.358 (1.150–1.604)	0.0003	
>100	163 (4.14%)	48 (41.03%)	18 (39.13%)	0.892 (0.375–2.122)	0.7966	
**Proteinuria**						0.869
Negative	3620 (95.21%)	634 (26.98%)	410 (32.28%)	1.347 (1.138–1.594)	0.0005	
Suspicious/Positive	182 (4.79%)	56 (50.00%)	35 (50.00%)	1.337 (0.663–2.695)	0.4169	


*^∗^p for interaction was test by likelihood ratio test.*

## Discussion

Our study identified significant association between periodontal disease and hypertension risk, and such association remained significant after adjustment for several potential confounding factors and propensity score adjusted analysis. These results were in line with those from some previously studies (Martin-Cabezas et al., 2016) in Asian populations. Lee et al. (2015) indicated that periodontitis significantly increased hypertension risk by 1.07 times in Koreans (OR = 1.07, 95%CI = 1.05–1.08), Kawabata et al. (2016) presented significantly increased risk of hypertension in Japanese (OR = 2.07, 95%CI = 1.19–6.29), and Zainoddin et al. (2013) showed similar tendency in Malaysian (OR = 1.99, 95%CI = 1.00–3.85). While in the current study, we used both multivariate adjustments and propensity score adjustment; therefore, we could isolate independent relations between periodontal disease and hypertension.

In our study, higher risk of developing hypertension was revealed in unadjusted periodontitis group (OR = 1.81), age <40 years group (OR = 1.69), LDL-C abnormal group (OR = 1.58) and ESR abnormal group (OR = 2.09). The magnitude of such relationship was comparable with that in previous research measuring well described risk factors for hypertension such as abdominal obesity (OR = 1.51, 95%CI = 1.27–1.81; Ostchega et al., 2012). Besides, our study also investigated the influence of BMI on the association between periodontal disease and hypertension risk, and also observed significantly positive relationship in both normal and abnormal BMI groups, suggesting the lack of interactions between periodontal disease and BMI. Interestingly, we also found an influence of age on the association between periodontal disease and hypertension; specifically, the relationship between two diseases was statistically significant in age <40 years group (OR = 1.69), but such significance weakened and even disappeared with age (aged 40–60 years: OR = 1.27; age ≥60 years: OR = 1.01; Figure 1). Previous evidence has manifested that abnormal FBG [including impaired fasting glucose (IFG) and diabetes mellitus (DM)] was an independent risk factor for hypertension (Morio et al., 2013), and our results further suggested that FBG status might also affect the association between periodontal disease and hypertension, because such association was significant in normal FBG but non-significant in abnormal FBG. However, all above mentioned interaction tests got non-significant results, implying such phenomenon might be attributed to insufficient sample size.

To our knowledge, periodontal disease has already been reportedly linked to several systemic diseases/conditions such as stroke (Lafon et al., 2014), oral cancer (Zeng et al., 2013), lung cancer (Zeng et al., 2016b), pancreatic cancer (Maisonneuve et al., 2017), erectile dysfunction (Cheraghi and Doosti-Irani, 2017), bladder cancer (Xie et al., 2018), inflammatory bowel disease (Papageorgiou et al., 2017), and gestational DM (Abariga and Whitcomb, 2016) showing the impact of periodontal health at systemic level. As for potential mechanism of periodontal disease involved in diseases, several hypotheses have been proposed, including endothelial dysfunction, oxidative stress and worsening of systemic inflammation in response to bacteremia, and inflammatory mediator dissemination from periodontal lesion (Leong et al., 2014; Macedo Paizan and Vilela-Martin, 2014; Martin-Cabezas et al., 2016). In our interaction analysis, only one significant interaction was observed, one between periodontal disease and ESR (Figure 1). As we know, both ESR and CRP reflect the degrees of inflammation; hence, in the presence of abnormal ESR, the risk of hypertension associated with periodontal disease was significantly increased from 1.28 to 2.09; while facing abnormal VRP, the risk of hypertension related to periodontal disease was raised from 1.28 to 1.78. This means that the presence of periodontal disease simultaneously accompanied by other inflammation would elevate the risk of hypertension, proving inflammation theory from the perspective of systemic cytokines modulation (Buhlin et al., 2009).

According to the evidence from our study here, the association exists between periodontal disease and increased hypertension risk; however, cross-sectional design cannot identify causation. Therefore, the following question we face is whether this association is causal. We can now examine the argument for causation based on Hill’s criteria (Bird, 2011). First, there was no clear temporal relationship in this study; hence, we did not know whether periodontal disease preceded the onset of hypertension, or reversed. Second, although the total sample size was larger and the CIs were narrow, all adjusted OR values did not surpass 1.50. A recent meta-analysis (Martin-Cabezas et al., 2016) suggested that periodontal disease significantly increased the risk of hypertension by 1.50 times (OR = 1.50, 95%CI = 1.27–1.78); however, this meta-analysis enrolled no study based on Chinese population, though our results were similar to those in the meta-analysis. Considering genetic background, such result might be not proper to representing general Chinese population. Third, periodontal disease can be categorized in minor, moderate, and severe types. The worse is we could not conduct dose-response analysis owing to insufficient data. However, a meta-analysis summarizing interventional mechanistic studies focusing on biomarkers associated with cardiovascular disease outcomes indicated that periodontal treatment improved cardiovascular diseases outcomes (D’Aiuto et al., 2013), still with limitations of lacking Chinese population data and not concerning on hypertension. Fifth, underlying mechanism of the association between periodontal disease and hypertension is inflammation, showing biological plausibility (Schenkein and Loos, 2013). Finally, as noted previously, after adjusting confounding factors and applying propensity score adjusted model, final results were still significant.

As we know, diseases, especially hypertension, might be influenced by genetic background. In Chinese hypertensive individuals, the prevalence of H-type Hypertension was more than 75% dramatically different from that in Europeans (Qin and Huo, 2016). Hence, it is important to investigate the association between periodontal disease and hypertension among Chinese population. Our study provided evidence supporting such relationship between two diseases; besides, unlike previous publications, our study adopted propensity score adjusted method, as well as interaction and subgroup analyses to detect moderator effects. However, some limitations in this study should be noted. Since the data adopted in our study were from previous health examinations in one university, we cannot obtain information on smoking status. Smoking is a shared risk factor for both periodontal disease and hypertension (Tsioufis et al., 2011), and missing its information might produce certain bias into our findings, though this limitation might generate less influence considering the fact that smoking is possibly unusual among the university staff and workers. Nonetheless, just as every coin has two sides, similar educational and socioeconomic status among this group of people also contributed to the risk of periodontal disease and hypertension (Tsioufis et al., 2011). Second, the collected data only reported periodontal status without detailed data on every participant; therefore, we could not analyse the CPITN score in the normotensive and hypertensive group, respectively. Besides, the degree of periodontitis (mild, moderate, and severe) cannot be analyzed due to the lack of detailed information either, while earlier studies have indicated that moderate to severe periodontitis led to higher risk of hypertension (Martin-Cabezas et al., 2016). Third, just like the above-mentioned limitations, intelligence about the history of hypertension or taking medicines for hypertension was not obtained either. If hypertensive cases had taken anti-hypertensive drugs before blood pressure measurement, their blood pressures would usually show normal levels; hence, we speculated that the number of individuals actually having hypertension might be large than that we counted in analysis. In other words, the risk of undervaluation might exist; however, our results still revealed a significantly increased risk of hypertension associated with periodontal disease.

In summary, the current study uncovered positive relationship between periodontal disease and hypertension risk in a Chinese university population. That is, periodontal disease patients would face higher risk of developing hypertension when compared with individuals having healthy periodontium. It has been emphasized that poor oral health might have a direct relationship with hypertension onset, which offers important implications for clinical practice and public health. And it is critical to investigate whether periodontal disease is causally linked to hypertension in a longitudinal setting, and whether dental therapy currently used in clinical practice can reduce hypertension risk, as well as the exact mechanisms of this association.

## Ethics Statement

This study was reviewed and approved by the Committee for Ethical Affairs of the Huaihe Hospital of Henan University, Henan Province (Approval No. 2018068).

## Author Contributions

M-JZ and X-TZ designed this study. Y-XQ and B-HL collected the data. M-JZ re-checked the data. QH performed the analysis. X-TZ re-checked analyses and reviewed the manuscript. M-JZ and LW wrote the manuscript.

## Conflict of Interest Statement

The authors declare that the research was conducted in the absence of any commercial or financial relationships that could be construed as a potential conflict of interest.
